# Effect of liraglutide on blood pressure: a meta-analysis of liraglutide randomized controlled trials

**DOI:** 10.1186/s12902-018-0332-5

**Published:** 2019-01-07

**Authors:** Xu Zhao, Kun Huang, Meijie Zheng, Junting Duan

**Affiliations:** 1 Civil Aviation General Hospital, Peking University, Beijing, China; 2 Endocrinology Department, Civil Aviation General Hospital, Chaoyang Road, Beijing, China

**Keywords:** Blood pressure, Cardiovascular risk factor, DBP, GLP-1RAs, Liraglutide, SBP

## Abstract

**Background:**

Several clinical trials have studied the effects of glucagon-like peptide-1 receptor agonists (GLP-1RAs) on glycometabolism and cardiovascular risk factors since they were identified. Because of their cardiovascular benefits and efficacy in lowering glucose, GLP-1RAs are becoming increasingly important in clinical therapy for patients with or without pathoglycaemia. The aim of this study was to assess the effect of the GLP-1RA liraglutide on blood pressure based on randomised controlled trials (RCTs).

**Methods:**

We searched PubMed for RCTs published from 2009 to 2018 comparing the effect of liraglutide on blood pressure with that of placebo in individuals with or without pathoglycaemia. RCTs in humans that included data describing blood pressure changes from baseline to the end of the trial were selected for inclusion in the meta-analysis.

**Results:**

A total of 18 RCTs that enrolled 7616 individuals in the liraglutide group and 6046 individuals in the control group were included in this meta-analysis. Compared with placebo, liraglutide reduced systolic blood pressure (SBP) by 3.18 mmHg (95% CI -4.32, − 2.05), *P* < 0.00001, but had no significant effect on diastolic blood pressure (DBP). Subgroup analysis showed that the degree of reduction in SBP was associated with the dose of liraglutide but that significance disappeared when the intervention lasted over 1 year. Liraglutide 3.0 mg/d significantly reduced DBP by 1.46 mmHg (95% CI -2.61, 0.32), *P* = 0.01, but liraglutide 1.8 mg/d slightly increased DBP by 0.47 mmHg (95% CI 0.11, 0.83), P = 0.01, compared with placebo.

**Conclusions:**

This meta-analysis demonstrated that liraglutide significantly reduced SBP in individuals with or without pathoglycaemia compared with placebo, but the difference was no longer significant when the intervention lasted over 1 year. Moreover, the effect of liraglutide on blood pressure is associated with the dose. This finding may provide additional evidence for cardiovascular protection.

## Background

Diabetes, a chronic and progressive metabolic disorder, is becoming a public health issue with a high prevalence and serious complications. The IDF (International Diabetes Federation) has estimated that there will be 59,200,000 patients suffering from diabetes in 2035 [1]. Long-term hyperglycaemia leads to macrovascular and microvascular complications, which places a heavy burden on the health care system [2]. Diabetes, especially type 2 diabetes, is associated with overweight/obesity, hypertension and dyslipidaemia. As a result, the American Diabetes Association (ADA) has recommended providing the components of diabetes care with the cardiovascular risk factors included [3]. Many large clinical studies have confirmed that blood pressure is one of the cardiovascular risk factors associated with diabetes, and strict blood pressure control could improve the cardiovascular prognosis of diabetic patients [4–7]. According to the ADVANCE study, a reduction of 5.6 mmHg in SBP could significantly reduce the relative risk of death from cardiovascular disease by 18% [8].

GLP-1 is an endogenous incretin secreted by the intestines after eating and can promote the secretion of insulin, inhibit the secretion of glucagon, delay gastric emptying, and maintain the stability of blood glucose. Based on this activity, GLP-1RAs, which decrease glucose, the risk of hypoglycaemia and weight, have been developed and used in the treatment of type 2 diabetes patients. GLP-1RAs have been shown to have either superior or noninferior efficacy compared with other hypoglycaemic agents, such as metformin, thiazolidinediones (TZDs), insulin, sulfonylureas, and dipeptidyl peptidase-4 (DPP-4) inhibitors [9–16]. In addition, some studies confirmed that GLP-1RAs could significantly reduce weight, improve insulin sensitivity [17–22], and protect the function of β-cells [23, 24].

In recent years, an increasing number of studies have suggested that GLP-1RAs might produce further benefits with regard to cardiovascular factors [25, 26]. Initially, Viswanathan et al. found that adding exenatide treatment to existing insulin therapy in patients with type 2 diabetes could significantly reduce blood pressure by 9.2 mmHg from baseline and that the reduction in blood pressure was independent of weight loss [27]. Since then, several research teams have conducted clinical studies investigating the efficacy of GLP-1RAs on blood pressure and other cardiovascular risk factors with different conclusions. Most studies concluded that GLP-1RAs could significantly reduce SBP and had a tendency to reduce DBP. Rosso et al. found that SBP significantly decreased by 14.7 mmHg and that DBP significantly decreased by 9 mmHg after 12 months of treatment with liraglutide, while fasting blood glucose, HbA1C, weight, waist circumference, and lipid levels also decreased significantly [28]. A study in nondiabetic obese adults found that SBP decreased by 5.7 mmHg (1.2 mg/day), 5.6 mmHg (1.8 mg/day), 8.8 mmHg (2.4 mg/day), and 6.9 mmHg (3.0 mg/day) compared with baseline after a 20-week treatment with liraglutide and that DBP decreased by 1.2 mmHg, 1.8 mmHg, 1.4 mmHg and 2.9 mmHg, respectively [29]. The LEADER trial found that SBP decreased by 1.2 mmHg and that DBP increased by 0.6 mmHg in the liraglutide group after an intervention of 3.5 years [30, 31]. A study in diabetic patients on peritoneal dialysis found that SBP decreased by 20–30 mmHg after a 12-month treatment with liraglutide, which might be associated with instability of the patients’ volume load [32]. Therefore, exploring the influence of GLP-1RAs on blood pressure in a large population by collecting the data from all relevant trials is necessary.

Liraglutide is one of the long-acting GLP-1RAs marketed in Europe in 2009 and has better efficacy with regard to cardiovascular benefits and hyperglycaemia reduction [11, 33, 34]. This meta-analysis aimed to investigate the effect of liraglutide on blood pressure in individuals with abnormal glucose metabolism or metabolic syndrome by searching randomised controlled trials (RCTs).

## Methods

The main objective of this meta-analysis was to assess the influence of liraglutide on blood pressure compared with that of placebo. Outcome measurements included SBP and DBP. We followed the methods specified in the Cochrane Handbook for Reviews on Interventions [35].

### Search strategy

Eligible trials were identified by electronic and manual searches. Electronic searches were conducted by searching PubMed for articles dating from 2009 to 2018 using the terms “liraglutide” and “blood pressure”. Manual searches were performed by reading the title, abstract and full text of relevant articles.

### Study selection

After searching for candidate articles, further identification of these articles was based on the inclusion and exclusion criteria described below. The process was performed independently by two investigators.

The inclusion criteria were as follows: (a) published studies in humans; (b) randomised, placebo, parallel controlled trials; (c) outcome measurements included blood pressure.

The exclusion criteria were as follows: (a) participants suffered from severe liver or renal insufficiency and required replacement therapy; (b) cross-over control trials; or (c) using diuretics or drinking too much water, which might impact volume load.

### Data extraction

The main data were extracted from each study after a full-text reading of each RCT included in the meta-analysis and included the following: (a) general information, such as the first author, title, year of publication, and sample size; (b) baseline characteristics of participants, such as age and duration of diabetes; (c) intervention measures, the duration of intervention and background therapy; and (d) changes in SBP and DBP from baseline to endpoint with the format of the mean (standard deviation).

### Quality assessment

The quality assessment of these RCTs included in the meta-analysis was performed according to the Cochrane Collaboration’s risk of bias assessment tools, which included six parts: selection bias (random sequence generation and allocation concealment), performance bias (blinding of participants and personnel), detection bias (blinding of outcome assessment), attrition bias (incomplete outcome data), reporting bias (selective reporting) and other bias [35].

### Data analysis

Statistical analysis was conducted by Review Manager (RevMan version 5.3). We assessed the heterogeneity among RCTs by using the Cochrane Q test and I^2^ statistic. I^2^ values of less than 25%, 25–50%, 50–75% and more than 75% represent no heterogeneity, mild heterogeneity, moderate heterogeneity and considerable heterogeneity, respectively. We concentrated on the changes in SBP and DBP from baseline to endpoint with the format of the mean (standard deviation). If the article did not provide a calculated standard deviation, we imputed it via sample size, standard error, 95% confidence interval and *p* value. The results of the meta-analysis were expressed as the weighted mean difference with 95% confidence intervals. To increase the efficacy of the results, even if the heterogeneity was low or there was no heterogeneity, a random-effects model was selected.

### Compliance with ethics guidelines

This article is based on previously conducted studies and does not contain any studies with human participants or animals performed by any of the authors.

## Results

### Literature searches and study inclusion

**Fig. 1:**
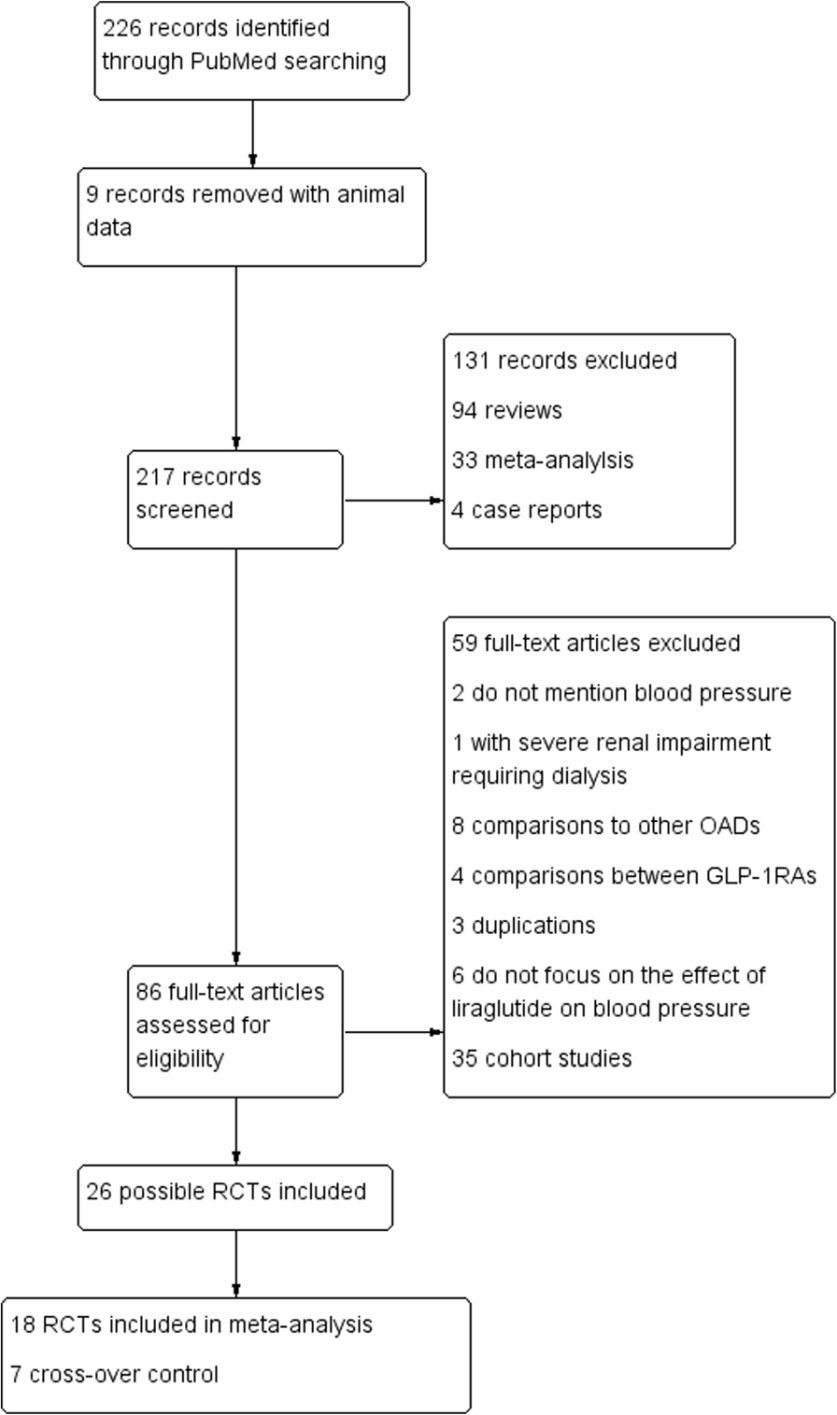
Study flow diagram

By searching PubMed, a total of 226 articles were screened. After excluding the articles that did not meet our inclusion criteria, 18 RCTs were included in the data analysis. All the included studies were randomised, double-blind, placebo, and parallel controlled trials. The study flow diagram is shown in Fig. 1. The characteristics of the RCTs included in the meta-analysis are shown in Table 1 and Table 2. Liraglutide was given at 0.6 mg once daily in 2 trials [36, 37]. As liraglutide at 0.6 mg/d is rarely used in clinical practice, we removed these data from the comparison of liraglutide with placebo.

**Table 1:** Summary of the RCTs included in the meta-analysis [29, 31, 36–51]

Study	Phase of the study	Duration of intervention	Background therapy	Background disease	Sample size	Intervention group	Measurement of BP	Antihypertensive pharmacological therapy
A Ahmann 2015	Phase 3	26-week	Insulin, metformin	T2DM	451	Liraglutide 1.8 mg/d (*n* = 225)	Collected at trial visit, without detail	Stable drug dose for at least 8 weeks prior to inclusion and throughout the trial^Φ^
A Astrup 2009	Phase 2	20-week	Diet, exercise	Metabolic syndrome	564	Liraglutide 1.2 mg/d (n = 85), 1.8 mg/d (n = 74), 2.4 mg/d (n = 73), 3.0 mg/d (n = 82)	Standardised method [69, 70]	Continued their baseline antihypertensive therapies^Φ^
A Astrup 2012	Phase 2	1-year	Diet, exercise	Metabolic syndrome	564	Liraglutide 1.2 mg/d (*n* = 85), 1.8 mg/d (*n* = 74), 2.4 mg/d (*n* = 73), 3.0 mg/d (*n* = 82)	Standardised method [69, 70]	Continued their baseline antihypertensive therapies^Φ^
A Blackman 2016	Phase 3	32-week	Diet, exercise	Metabolic syndrome	359	Liraglutide 3.0 mg (*n* = 178)	Collected at trial visit, without detail	Stable drug dose for at least 3 months prior to inclusion and throughout the trial^Φ^
Christian 2015	Phase 3	12-week	Insulin	T1DM	40	Liraglutide 1.2 mg/d (*n* = 18)	Collected at trial visit, without detail	Continued their baseline antihypertensive therapies^Φ^
Dejgaard 2016	Phase 4	24-week	Insulin	T1DM	100	Liraglutide 1.8 mg/d (*n* = 46)	Using a portable device (Spacelab Medical Model 90,217, Deerfield, WI, USA) with an appropriately sized cuff around the non-dependent upper arm after excluding between-arm differences in blood pressure > 5 mmHg	Remained unchanged during the study period^Φ^
LEAD-1	Phase 3	26-week	Glimepiride 2–4 mg/d	T2DM	1041	Liraglutide 1.2 mg/d (*n* = 228)	Standardised method [69]	Continued their baseline antihypertensive therapies^Φ^
						Liraglutide 1.8 mg/d (*n* = 234)		
LEAD-2	Phase 3	26-week	Metformin 1 g bid	T2DM	1662	Liraglutide 1.2 mg/d (*n* = 241)	Standardised method [69]	Continued their baseline antihypertensive therapies^Φ^
						Liraglutide 1.8 mg/d (*n* = 242)		
LEAD-4	Phase 3	26-week	Metformin 1 g bid	T2DM	821	Liraglutide 1.2 mg/d (n = 178)	Standardised method [69]	Continued their baseline antihypertensive therapies^Φ^
			rosiglitazone 4 mg bid			Liraglutide 1.8 mg/d (n = 178)		
LEAD-5	Phase 3	26- 26- Week	Metformin 1 g bid	T2DM	581	Liraglutide 1.8 mg/d (*n* = 232)	Standardised method [69]	Continued their baseline antihypertensive therapies^Φ^
			glimepiride 4 mg/d					
LEADERS trial	Phase 3	3.8-year	Diet, exercise, OAH or insulin	T2DM	9340	Liraglutide 1.8 mg/d (*n* = 4668)	Standardised method [69]	Target: 130/80 mmHg; First line: ACE inhibitors or ARBs; Based on individual patient needs: Ca2+ blockers, diuretics, others
Mark M. Smits 2016	Phase 4	12-week	Metformin, sulphonylurea	T2DM	60	Liraglutide 1.8 mg/d (*n* = 19)	Standardised method using an automatic oscillometric device (Dinamap, GE Healthcare, Little Chalfont, UK) [71]	Continued their baseline antihypertensive therapies^Φ^
MDI liraglutide trial	Phase 2	24-week	Insulin	T2DM	124	Liraglutide 1.8 mg/d (*n* = 63)	Collected at trial visit, without detail	Remained unchanged during the study period^Φ^
Nandy 2014	Phase 3	12-week	Lifestyle, metformin	T2DM	49	Liraglutide 1.8 mg/d (*n* = 16)	An arterial catheter was placed in the non-dominant arm in series with a pressure transducer	Stable drug dose for at least 4 weeks prior to inclusion and throughout the trial^Φ^
P. Mensberg MSc 2016	Phase 4	16-week	Exercise	T2DM	33	Liraglutide 1.8 mg/d (n = 17)	Measured on the left arm after the patients had rested for 10 min	Remained unchanged during the study period^Φ^
Robert 2015	Phase 4	12-week	Diet, exercise	Metabolic syndrome	44	Liraglutide 1.8 mg/d (*n* = 21)	Not mentioned	Without hypertension
S Frossing 2018	Phase 4	26-week	Lifestyle	Metabolic syndrome	72	Liraglutide 1.8 mg/d (*n* = 48)	Standardised method [69, 72]	Without hypertension
Sun H. Kim 2013	Phase 3	14-week	Diet, exercise	Prediabetes	68	Liraglutide 1.8 mg/d (n = 24)	Standardised method using a Dinamap automatic blood pressure recorder (GE Healthcare, Tampa, FL)	Without hypertension

(Φ the number of subjects on antihypertensive pharmacological treatment was not mentioned; *BP* Blood pressure, *DM* Diabetes mellitus, *OAH* Oral antihyperglycaemics, *ACE inhibitors* Angiotensin-converting enzyme inhibitors, *ARBs* Angiotensin receptor blockers)

**Table 2:** Baseline Characteristics of trials included in the meta-analysis [29, 31, 36–51]

Study	Age (years)	Duration of diabetes (years)	BMI (kg/m^2^)	Hypertension (n,%)	SBP (mmHg)	DBP (mmHg)
A Ahmann 2015	I:59.3(9.2)	I:12.1(7.1)	I:32.3(5.6)	Unavailable	Unavailable	Unavailable
	P:57.5(11.1)	P:12.1(6.8)	P:32.2(5.7)			
A Astrup 2009	I:47.2(9.7)^†^	NA	I:34.8(2.6)^†^	Unavailable	I:127(13.1)^†^	I:79.7(9.1)^†^
	45.5(10.9)^‡^		35.0(2.6)^‡^		123(13.0)^‡^	77.9(7.9)^‡^
	45.0(11.1)^§^		35.0(2.8)^§^		126(13.9)^§^	78.6(8.2)^§^
	45.9(10.7)^♭^		34.8(2.8)^♭^		124(11.3)^♭^	77.8(8.3)^♭^
	P:45.9(10.3)		P:34.9(2.8)		P:124(11.1)	P:76.8(8.5)
A Astrup 2012	I:47.2(9.7)^†^	NA	I:34.8(2.6)^†^	Unavailable	I:127(13.1)^†^	I:79.7(9.1)^†^
	45.5(10.9)^‡^		35.0(2.6)^‡^		123(13.0)^‡^	77.9(7.9)^‡^
	45.0(11.1)^§^		35.0(2.8)^§^		126(13.9)^§^	78.6(8.2)^§^
	45.9(10.7)^♭^		34.8(2.8)^♭^		124(11.3)^♭^	77.8(8.3)^♭^
	P:45.9(10.3)		P:34.9(2.8)		P:124(11.1)	P:76.8(8.5)
A Blackman 2016	I:48.6 (9.9)	NA	I:38.9(6.4)	I:75,41.7%	I:125.8(11.5)	I:81.2(7.6)
	P:48.4(9.5)		P:39.4(7.4)	P:77,43%	P:127.1(12.3)	P:82.2(8.8)
Christian 2015	I:39.5(2.7)	I:18.33(2.0)	I:24.17(0.64)	Unavailable	I:129.4(2.5)	I:75.5(1.7)
	P:36.1(1.6)	P:19.56(1.6)	P:22.75(0.41)		P:127.3(2.2)	P:72.5(1.4)
Dejgaard 2016	I:47(13)	I:20(12)	I:30.3(3.5)	Unavailable	I:131(15)	I:82(9)
	P:49(12)	P:25(12)	P:29.8(3.1)		P:130(16)	P:81(8)
LEAD-1	I:57.7(9)^†^	I:6.7(4.0,10.7)*^†^	I:29.8(5.1)^†^	I:155,68%^†^	I:133(15)^†^	Unavailable
	55.6(10)^‡^	6.5(3.7,10.5)*^‡^	30.0(5.1)^‡^	163,69.7%^‡^	132(16)^‡^	
	P:54.7(10)	P:6.5(4.5,10.6)*	P:30.3(5.4)	P:74,64.9%	P:131(15.3)	
LEAD-2	I:57.0(9)^†^	I:7.0(5)^†^	I:31.1(4.8)^†^	Unavailable	I:132(14)^†^	I:80(10)^†^
	57.0(9)^‡^	8.0(5)^‡^	30.9(4.6)^‡^		131(14)^‡^	79(8)^‡^
	P:56.0(9)	P:8.0(6)	P:31.6(4.4)		P:135(16)	P:81(9)
LEAD-4	I:55.0(10)^†^	I:9.0(6)^†^	I:33.2(5.4)^†^	Unavailable	I:129(14.8)^†^	I:75.8(9.0)^†^
	55.0(11)^‡^	9.0(6)^‡^	33.5(5.1)^‡^		126(14.2)^‡^	75.2(8.4)^‡^
	P:55.0(10)	P:9.0(6)	P:33.9(5.2)		P:128(14.5)	P:76.2(9.2)
LEAD-5	I:57.6(9.5)	I:9.2(5.8)	I:30.4(5.3)	Unavailable	I:135(15.0)	I:80.8(9.1)
	P:57.5(9.6)	P:9.4(6.2)	P:31.3(5.0)		P:133(14.0)	P:80.4(9.3)
LEADERS trial	I:64.2(7.2)	I:12.8(8.0)	I:32.5(6.3)	Unavailable	I:135.9(17.8)	I:77.2(10.3)
	P:64.4(7.2)	P:12.9(8.1)	P:32.5(6.3)		P:135.9(17.7)	P:77.0(10.1)
Mark M. Smits 2016	I:62.8(6.9)	Unavailable	I:32.0(30.9–35.9)*	Unavailable	I:136.6(17.0)	I:77.0(5.4)
					P:137.6(14.9)	P:76.4(6.8)
	P:62.8(6.9)					
			P:30.8(28.9–31.5)*			
MDI liraglutide trial	I:63.7(8.2)	I:17.3(7.6)	I:33.7(4.3)	Unavailable	I:137.9(16.8)	I:73.5(12.7)
	P:63.5(7.7)	P:17.0(8.1)	P:33.5(4.0)		P:133.7(13.7)	P:74.9(8.5)
Nandy 2014	I:57.7(9)	I:5.3(4.1)	I:32.7(4.5)	Unavailable	Unavailable	Unavailable
	P:60.3(7.3)	P:8.4(4.6)	P:31.6(4.2)			
P. Mensberg MSc 2016	I:56.5(9)	I:6(5.2)	I:32.5(3.7)	Unavailable	I:136.4(11.0)	I:84.1(7.0)
	P:55.6(12)	P:3.7(3.3)	P:32.4(5.2)		P:136.2(8.9)	P:82.1(7.0)
Robert 2015	I:34(9)	NA	I:36.15(3.84)	0,0%	I:130(15)	I:76(11)
	P:34(9)		P:35.74(4.55)		P:133(17)	P:78(10)
S Frossing 2018	I:29.9(6.1)	NA	I:33.3(5.1)	0,0%	I:123(9)	I:79(8)
	P:29.9(6.1)		P:33.3(4.6)		P:124(9)	P:80(7)
Sun H. Kim 2013	I:58.0(7)	NA	I:31.9(2.7)	0,0%	I:127(10)	I:76(9)
	P:58.0(8)		P:31.9(3.5)		P:119(14)	P:75(8)

(Abbreviations: *BMI* Body mass index, *SBP* Systolic blood pressure, *DBP* Diastolic blood pressure, *NA* Not applicable, *I* Intervention group, *P* Placebo group, age and the duration of diabetes were expressed as mean(SD) unless otherwise noted, * median (25th and 75th percentile); † liraglutide 1.2 mg/d, ‡ liraglutide 1.8 mg/d, § liraglutide 2.4 mg/d, ♭ liraglutide 3.0 mg/d))

### Quality assessment

**Fig. 2:**
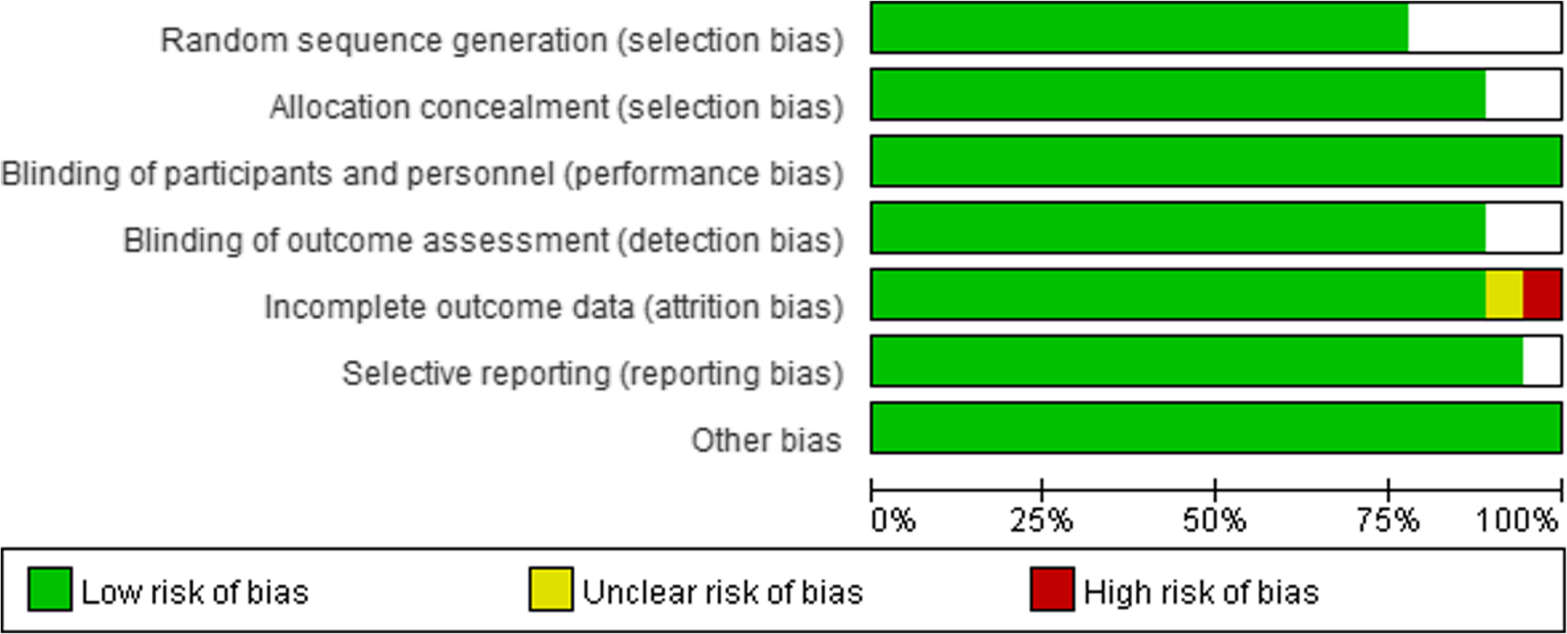
Risk of bias graph

**Fig. 3:**
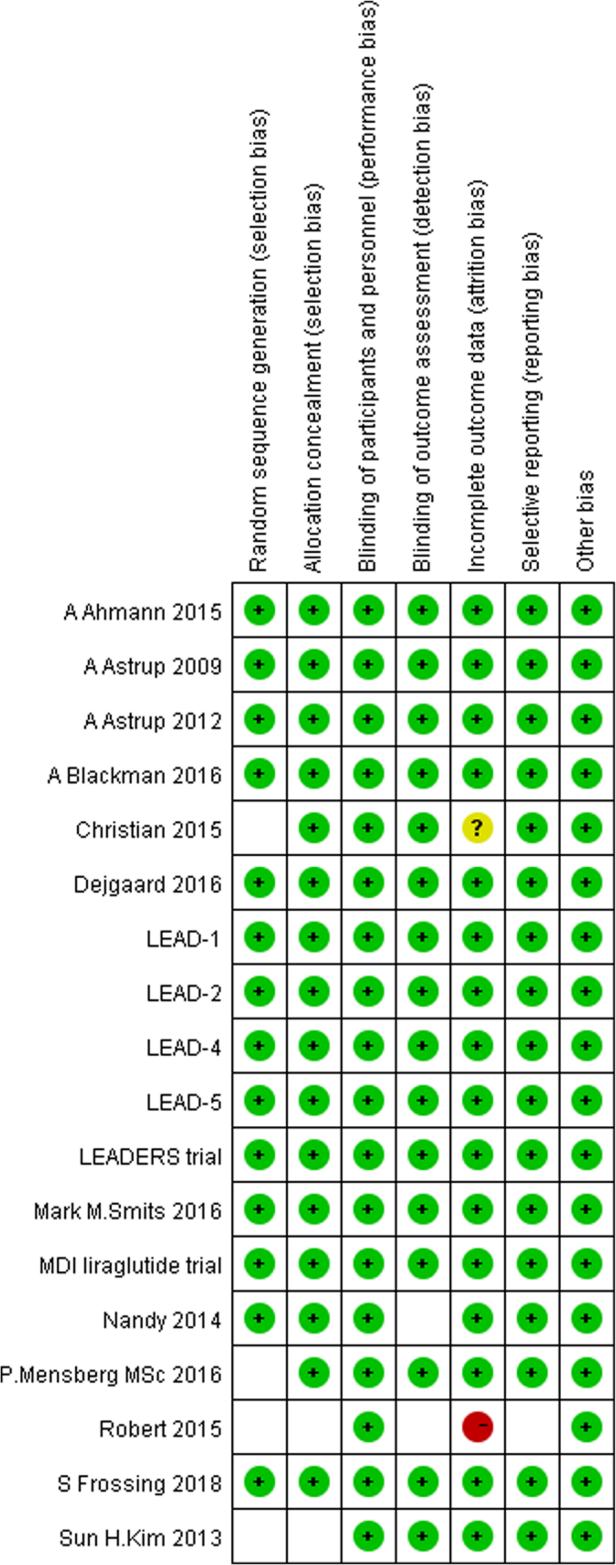
Risk of bias summary (+, low risk of bias; −, high risk of bias;?, unknown risk of bias)

We conducted a quality assessment of the 18 RCTs included in the meta-analysis according to the Cochrane Collaboration’s risk of bias assessment tools. The characteristics at baseline of all 18 RCTs showed no significant difference between the liraglutide group and the placebo group. Four RCTs did not provide clear information on random sequence generation and allocation concealment [38–41]. One RCT did not give the number of people who were lost to follow-up or withdrew and the reason [40]. All 18 RCTs were performed and assessed by blinding researchers and participants [29, 31, 36–51]. The risk of bias is shown in Fig. 2 and Fig. 3.

### SBP

**Fig. 4:**
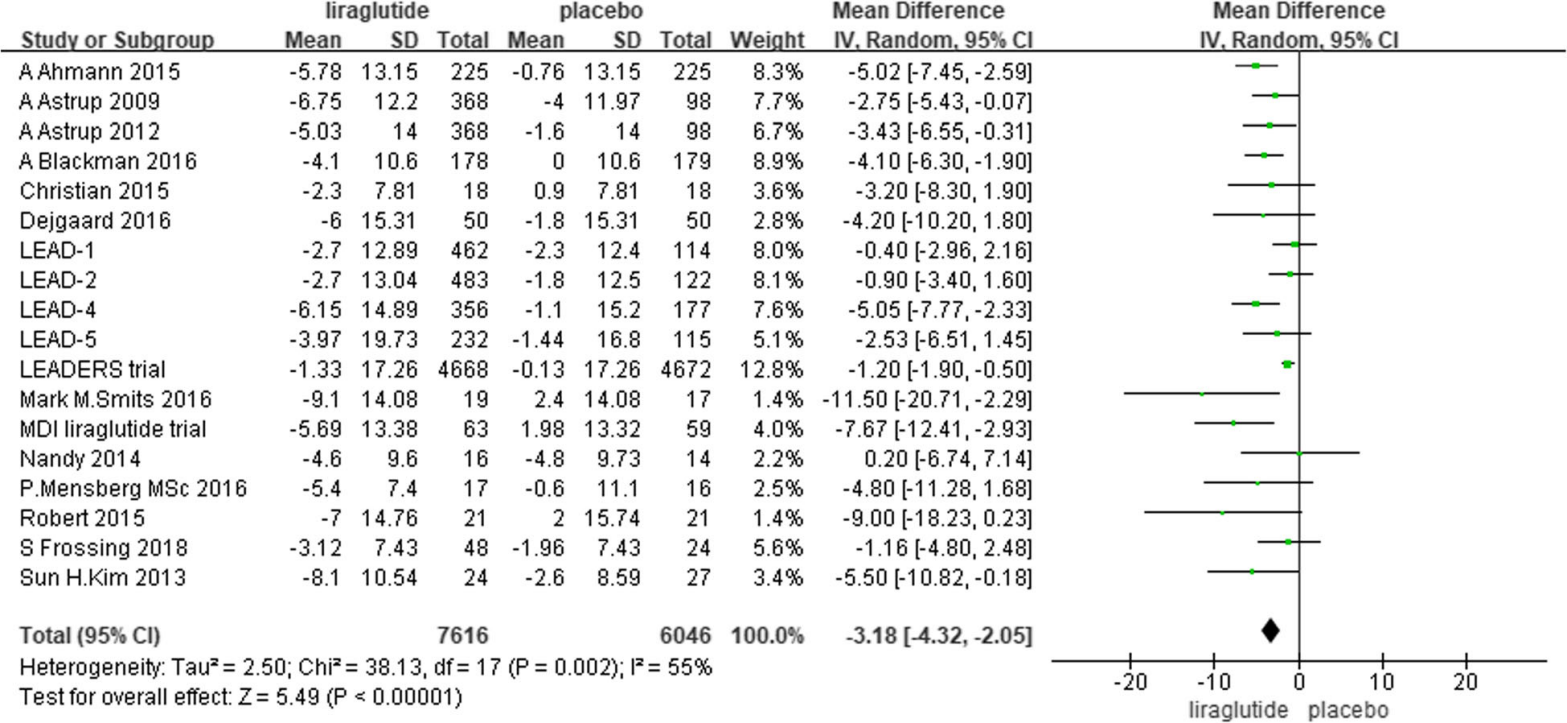
The forest plot of the comparison between the effects of liraglutide and placebo on SBP (random-effects model)

There were 7616 individuals in the liraglutide group and 6046 individuals in the placebo group included in the data analysis. Nine trials reported that liraglutide reduced SBP significantly compared with placebo [29, 31, 41–44, 46, 48, 49]. Eight trials did not show a significant difference in the reduction in SBP between liraglutide and placebo [36–40, 45, 47, 51], and 1 trial reported that liraglutide could slightly increase SBP without a clear significant difference [50]. The random-effects model showed that liraglutide significantly reduced SBP compared with placebo. The mean difference was 3.18 mmHg (− 4.32 to − 2.05, I^2^ = 55%, *P* < 0.00001) (Fig. 4). The I^2^ values suggested moderate heterogeneity, which might be related to the demographic characteristics, background therapy, dose of liraglutide and duration of intervention in each study.

**Fig. 5:**
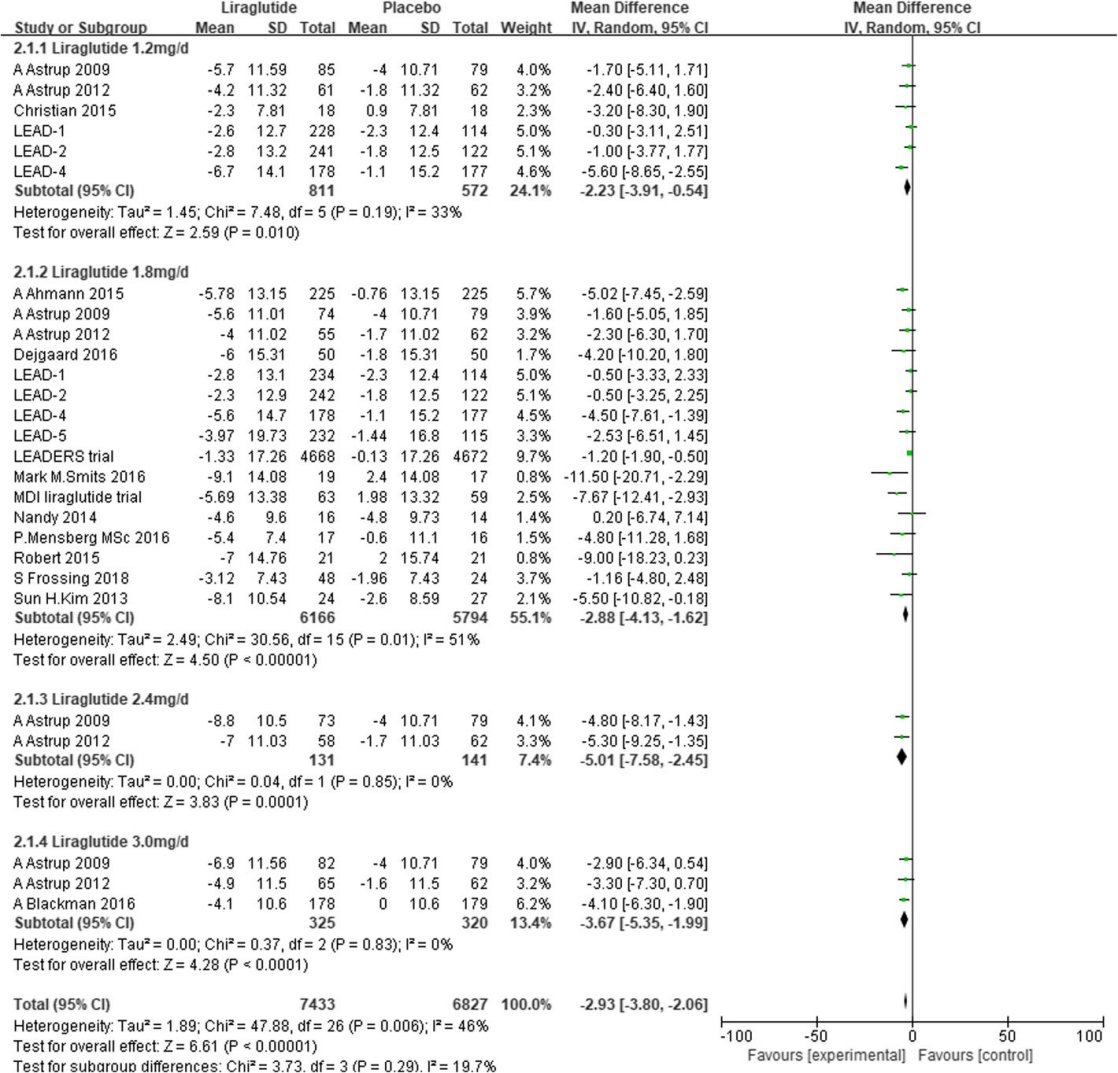
The forest plot of SBP in subgroup analysis defined by the dose of liraglutide (random-effects model)

We conducted subgroup analysis defined by the dose of liraglutide. Liraglutide significantly reduced SBP by 2.23 mmHg (− 3.91 to − 0.54, I2 = 33%, P < 0.00001), 2.88 mmHg (− 4.13 to − 1.62, I^2^ = 51%, P < 0.00001), 5.01 mmHg (− 7.58 to − 2.45, I^2^ = 0%, *P* = 0.0001), and 3.67 mmHg (− 5.35 to − 1.99, I^2^ = 0%, *P* < 0.0001) compared with placebo in the liraglutide 1.2 mg/d stratification, 1.8 mg/d stratification, 2.4 mg/d stratification and 3.0 mg/d stratification, respectively (Fig. 5).

**Fig. 6:**
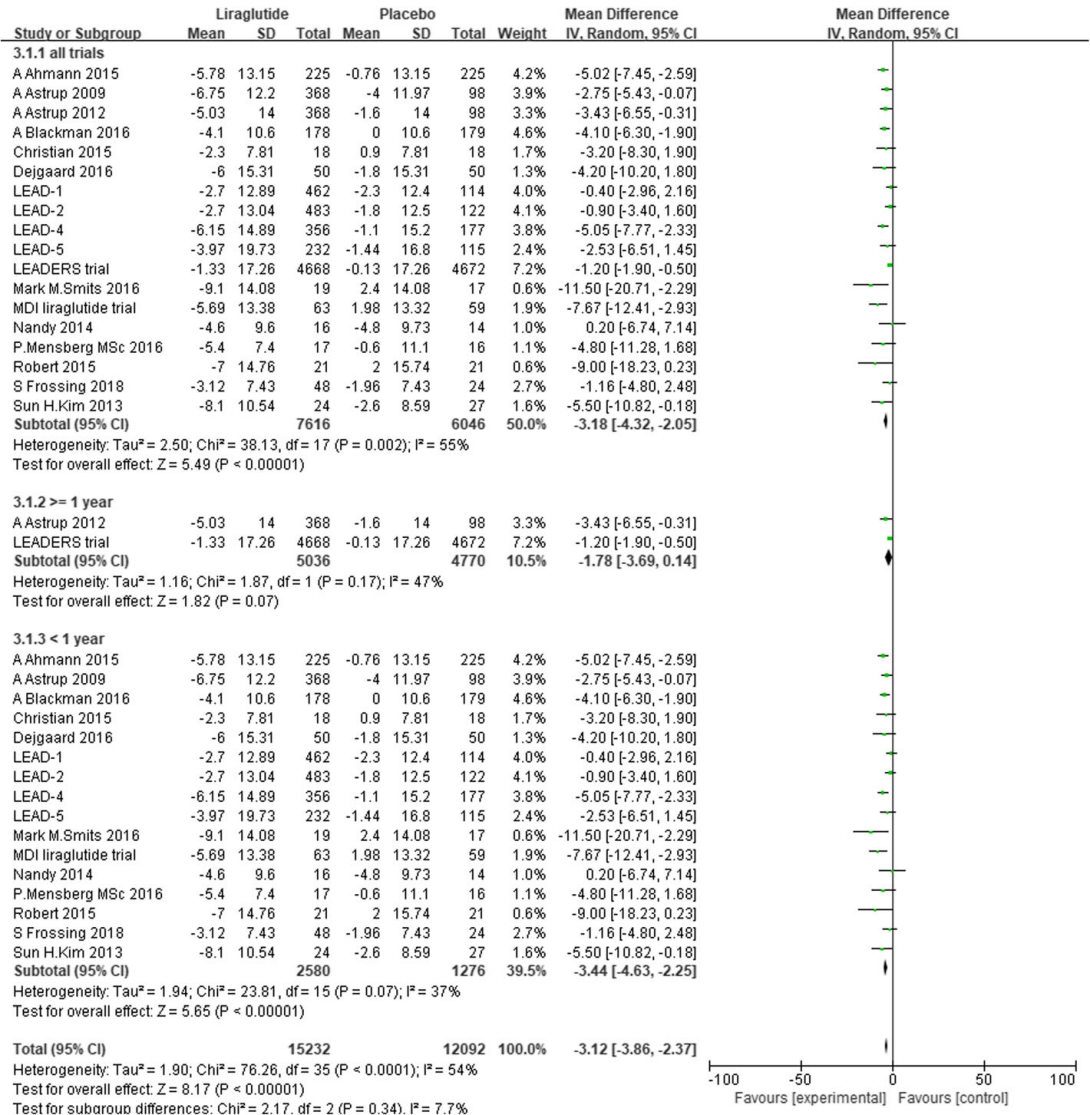
The forest plot of SBP in subgroup analysis defined by the duration of intervention (random-effects model)

In addition, we conducted subgroup analysis defined by the duration of intervention. Subgroup analysis did not show a significant difference in reduction in SBP between the liraglutide group with a more than 1-year duration of intervention and the placebo group. The mean difference was − 1.78 mmHg (− 3.69 to 0.14, *P* = 0.07, I^2=^47%). Compared with the placebo group, the liraglutide group with a less than 1-year duration of intervention showed a significant reduction in SBP of 3.44 mmHg (− 4.63 to − 2.25, *P* < 0.00001, I^2^ = 37%) (Fig. 6).

### DBP

**Fig. 7:**
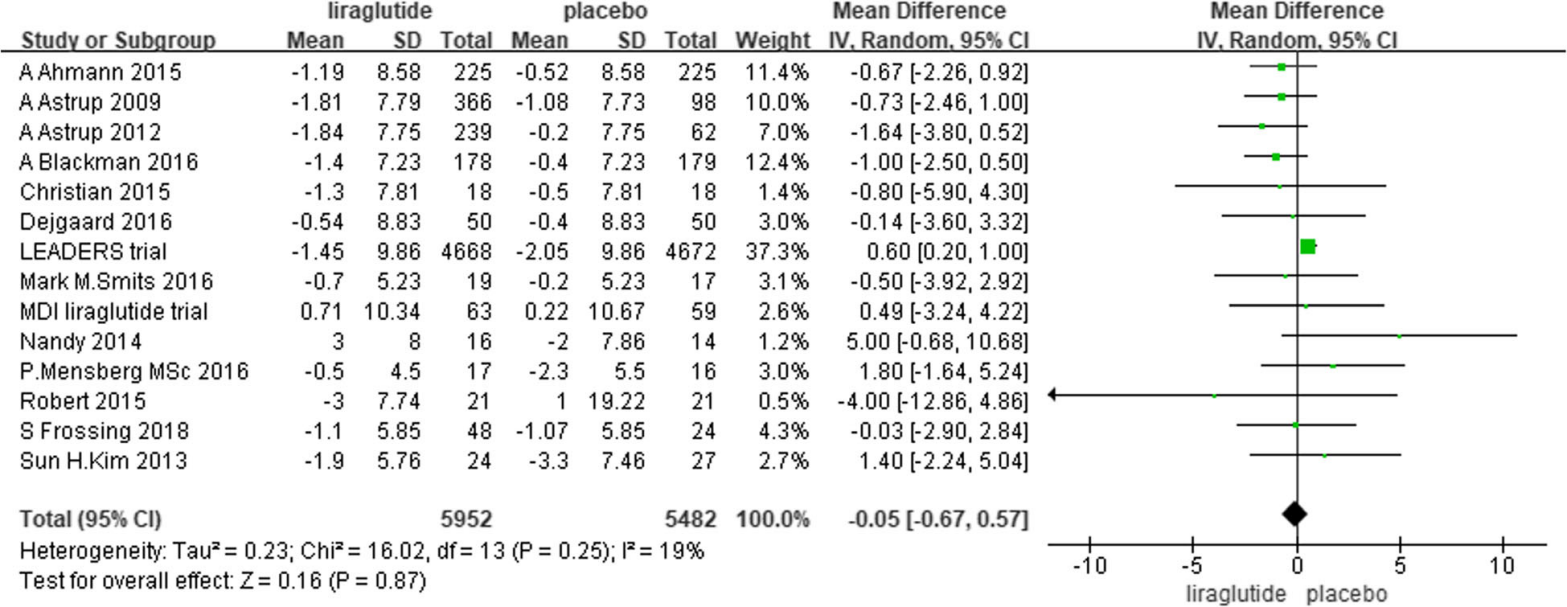
The forest plot of the comparison between the effects of liraglutide and placebo on DBP (random-effects model)

Fourteen trials reported changes in DBP from baseline to endpoint [29, 31, 38–45, 48–51]. We performed a random-effects meta-analysis with 5952 individuals assigned to liraglutide and 5482 individuals assigned to placebo. No significant difference was found in the reduction in DBP between liraglutide and placebo. The mean difference was − 0.05 mmHg (− 0.67 to 0.57, *P* = 0.87, I^2^ = 19%) (Fig. 7).

**Fig. 8:**
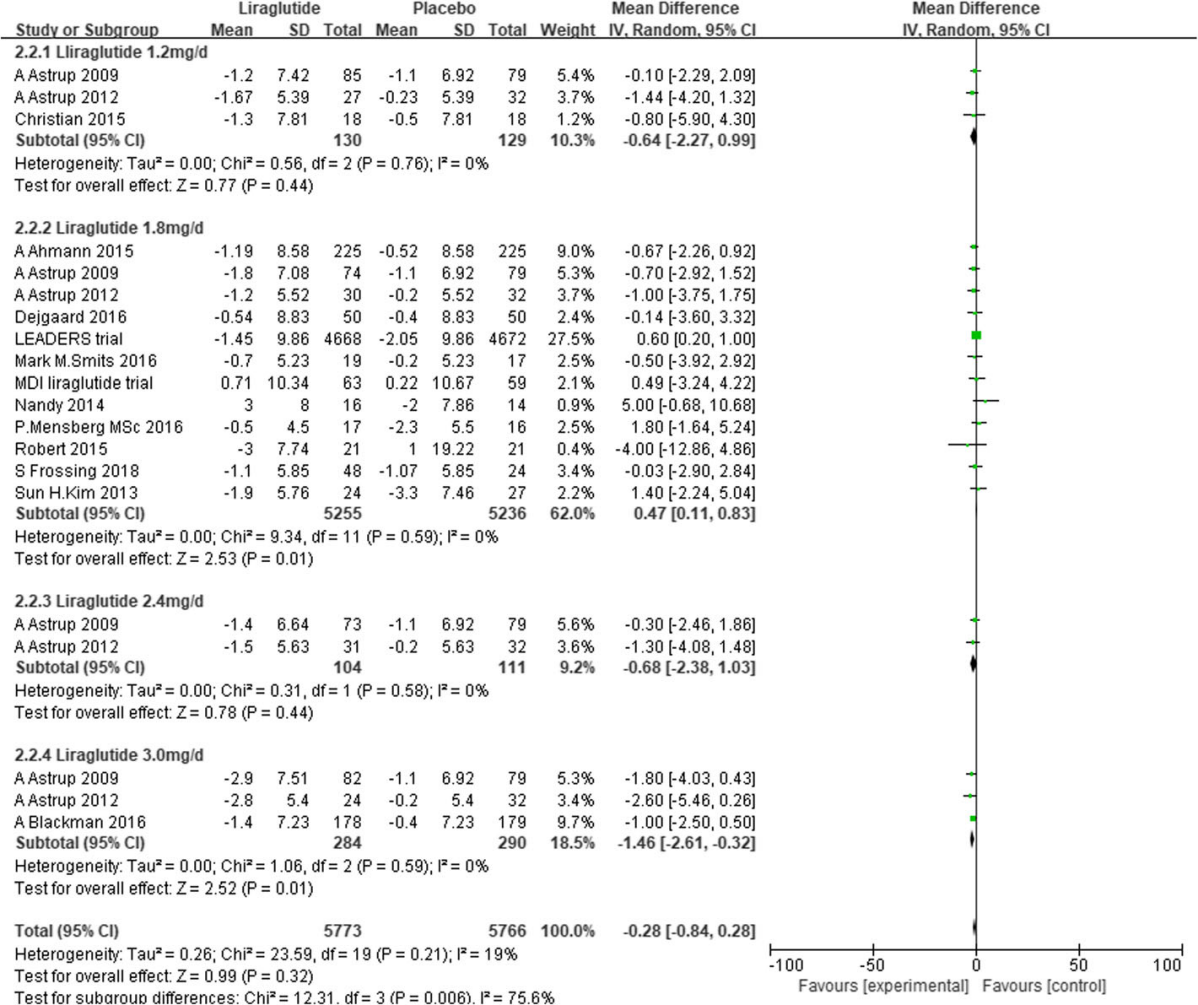
The forest plot of DBP in subgroup analysis defined by the dose of liraglutide (random- effects model)

**Fig. 9:**
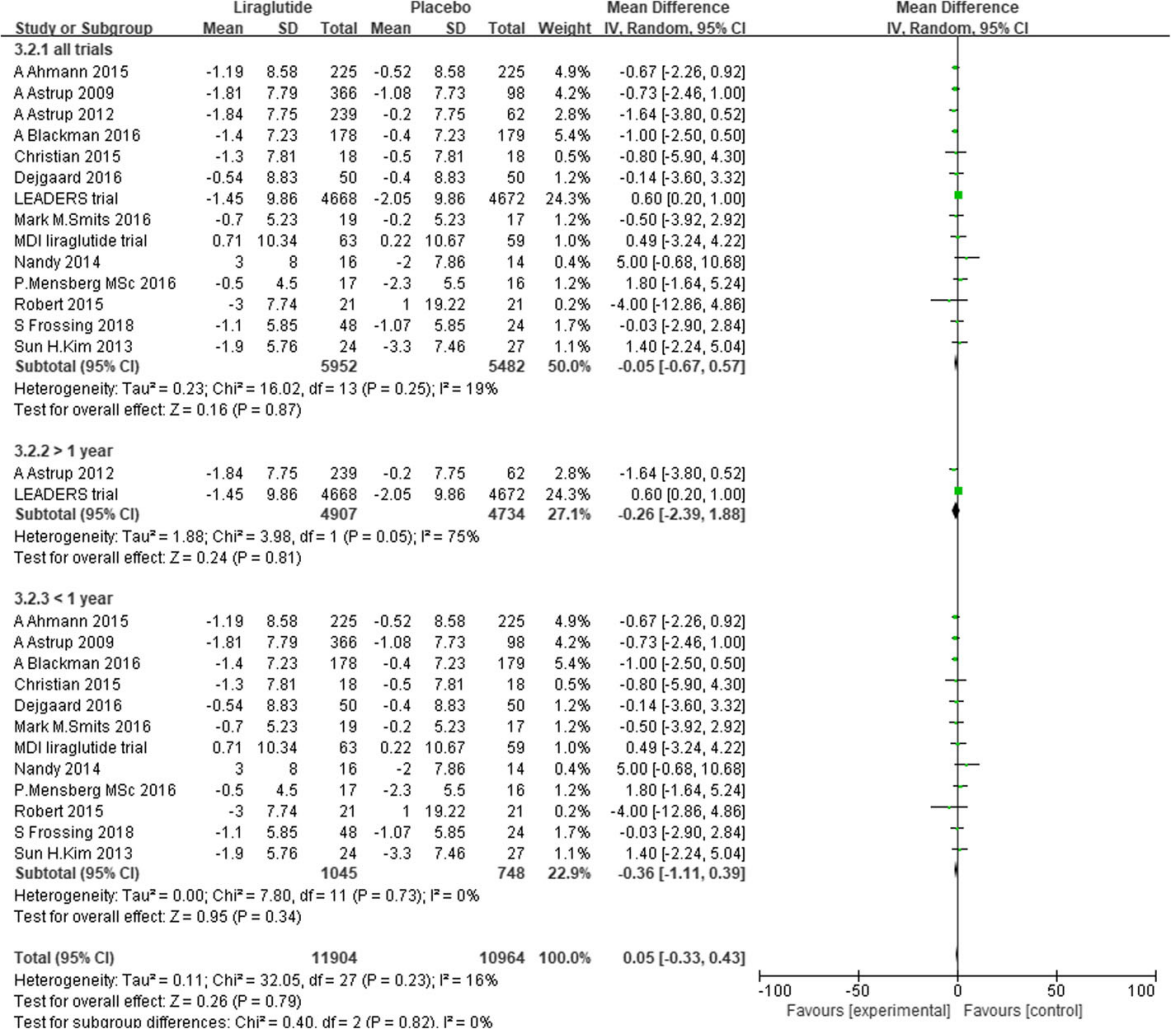
The forest plot of DBP in subgroup analysis defined by the duration of intervention (random-effects model)

We conducted subgroup analysis defined by the dose of liraglutide. Liraglutide 3.0 mg/d significantly reduced DBP by 1.46 mmHg (− 2.61 to − 0.32, I^2^ = 0%, *P* = 0.01) compared with placebo. However, liraglutide 1.8 mg/d slightly increased DBP by 0.47 mmHg (0.11 to 0.83, I^2^ = 0%, P = 0.01) (Fig. 8). In addition, we conducted subgroup analysis defined by the duration of intervention, which showed that liraglutide did not significantly reduce DBP compared with placebo, whether the duration of intervention was more than or less than 1 year (Fig. 9).

## Discussion

### Explanation for findings

As liraglutide 0.6 mg/d subcutaneous injection was well tolerated and rarely used in clinical practice, we eliminated the data on liraglutide 0.6 mg/d. The random-effects model showed a significant difference in reduction in SBP between liraglutide and placebo by − 3.18 mmHg but no significant difference in reduction in DBP. Subgroup analysis showed that the degree of reduction in SBP was associated with the dose of liraglutide and the duration of intervention. The larger the dose of liraglutide was, the greater the reduction in SBP. However, the degree of reduction in SBP declined when the dose of liraglutide was 3.0 mg/d. Subgroup meta-analysis showed that short-term intervention with liraglutide (less than 1 year) could reduce SBP significantly compared with placebo but that the difference in reduction would disappear when the intervention lasted over 1 year. The mechanism underlying this phenomenon was not clear. However, there were only 2 trials with a more than 1-year duration of intervention, so the results might be related to the adherence to the medication, or compliance of the participants. In addition, there were limited trials in the liraglutide 2.4 mg/d stratification and the liraglutide 3.0 mg/d stratification. Thus, additional long-term and large-dosage clinical trials are needed to probe the further efficacy of liraglutide on blood pressure.

### Assessment of quality of included studies

This meta-analysis included 18 RCTs. All of the included trials were randomised, double-blind, placebo-controlled, parallel trials. To improve the grade of evidence, we excluded cross-over controlled trials from comparisons between liraglutide and placebo. After quality assessment, the bias risk of the included trials was relatively low.

The GLP-1RA liraglutide, as a new method of antidiabetic therapy, has been shown by a considerable number of trials to demonstrate efficacy in lowering fasting blood glucose, postprandial blood glucose, and weight. Increasing numbers of clinical studies have shown its cardiovascular benefits, providing further evidence for clinical use of liraglutide beyond antihyperglycaemia [8–14, 17–20, 27, 28]. In recent years, some researchers performed meta-analyses to compare liraglutide and other antihyperglycaemic agents, such as sulfonylureas, insulin, TZDs, DPP-4 inhibitors and other GLP-1RAs, demonstrating different effects in lowering HbA1C/fasting plasma glucose/postprandial plasma glucose, adverse events, and improvement in insulin resistance, weight loss and the risk of hypoglycaemia [25, 26, 34, 52–57]. However, the influence of liraglutide on blood pressure was still uncertain.

Hypertension is highly correlated with diabetes but remains underrecognised and undertreated in the diabetic and the general population. The UK prospective diabetes study found that strict blood pressure control in patients with hypertension and type 2 diabetes substantially reduced the risk of death and complications due to diabetes [58]. In the active-treatment arm of the ADVANCE study, a decrease in blood pressure of 5.6/2.2 mmHg in high-risk patients with T2DM reduced the rate of renal adverse events by 21% [59]. Our meta-analysis showed that SBP was reduced by approximately 5 mmHg, which may be a cardioprotective benefit. A study based on healthy adults found that plasma levels of fasting GLP-1 are significantly and positively related to the blood pressure indices assessed [60]. The increase in GLP-1 levels could be a compensatory response to individual BP elevations. The possible mechanisms by which GLP-1 reduces BP are vasodilatory properties [61] and improvement of endothelial function [62, 63]. In addition, there is some evidence that GLP-1RAs mediate sodium excretion and diuresis in order to lower blood pressure [64–66].

Intensive control of glucose levels and blood pressure is currently the mainstay of both prevention and treatment of diabetic nephropathy. The LEADER trial showed that liraglutide-induced benefits on renal outcome could be due to improvements in renal risk factors, such as renal haemodynamics [67]. GLP-1RAs may induce renoprotection by inhibiting renal tubular sodium reabsorption, facilitating water excretion [64–66] and decreasing glomerular pressure. A pooled analysis of four studies showed that DPP-4 inhibitors led to a significant reduction in albuminuria in patients with type 2 diabetes [68].

All trials found that liraglutide significantly reduced body weight compared with placebo [29, 31, 36–51]. The reduction in SBP partly contributes to the reduction in body weight. However, on the basis of the SBP and weight profiles over time, the reduction in SBP may not be fully explained by the reduction in body weight [37]. Based on the time course of SBP and weight reductions, the reduction in SBP occurred before substantial weight loss [47]. A meta-analysis showed that significant reductions in SBP were observed as early as 2 weeks after initiation of liraglutide treatment and could be observed before any significant weight loss occurred [68].

### Strengths and limitations

The aim of this meta-analysis was to discuss the influence of liraglutide on blood pressure in individuals with or without abnormal glucose metabolism by searching high-quality RCTs to provide reliable evidence for clinical practice. However, some limitations should be noted. First, Robert SA et al. did not provide the number of people who were lost to follow-up or withdrew and the reasons these participants were lost follow-up or withdrew. Four RCTs did not give a clear method of random sequence and allocation concealment [38–41]. These factors increased the bias risk of the studies included. Second, because of the limitation of sample size in stratifications treated with liraglutide 2.4 mg/d and 3.0 mg/d, the subgroup analysis might be inaccurate. Third, there was a lack of clinical trials on the efficacy of liraglutide on blood pressure in patients with and without hypertension.

## Conclusions

In this meta-analysis, 18 RCTs were included to explore the effect of liraglutide on blood pressure. The results showed that compared with placebo, liraglutide significantly reduced SBP. At doses of liraglutide up to 3.0 mg/d, the reduction in DBP was significant. At present, liraglutide is widely recognised to have a beneficial effect on glucose reduction, weight loss and protection of β-cells. With the efficacy on blood pressure, the application of liraglutide in clinical practice may be broadened in the future. More clinical trials are needed to investigate the further effect of liraglutide on blood pressure.

## Abbreviations

ACE inhibitors: Angiotensin-converting enzyme inhibitors
ADA: American Diabetes Association
ARBs: Angiotensin receptor blockers
BMI: Body mass index
BP: Blood pressure
DBP: Diastolic blood pressure
DM: Diabetes mellitus
DPP-4: Dipeptidyl peptidase-4
GLP-1RAs: Glucagon-like peptide-1 receptor agonists
IDF: International Diabetes Federation
NA: Not applicable
OAH: Oral antihyperglycaemics
RCTs: Randomised controlled trials
SBP: Systolic blood pressure
TZDs: Thiazolidinediones
